# The effect of telerehabilitation on activity performance and participation in daily life in children with developmental coordination disorder: A randomized controlled trial

**DOI:** 10.1371/journal.pone.0330846

**Published:** 2025-08-21

**Authors:** Mazlum Uruc, Burak Menek

**Affiliations:** 1 Department of Occupational Therapy, Institute of Health Sciences, Istanbul Medipol University, Istanbul, Turkey; 2 Department of Physiotherapy and Rehabilitation, Istanbul Medipol University, Beykoz, Istanbul, Turkey; University of Chile: Universidad de Chile, CHILE

## Abstract

**Background:**

Developmental Coordination Disorder (DCD) is a neurodevelopmental condition that adversely impacts motor skills, sensory processing, and daily activity participation. Telerehabilitation has recently emerged as a promising method to improve therapy access and foster family involvement. This study investigated the effects of integrating telerehabilitation with sensory-based intervention on motor performance, sensory processing, and participation in children with DCD.

**Methods:**

This randomized controlled trial included 20 children aged 3–7 years with a confirmed diagnosis of DCD. Participants were randomly assigned to either a sensory-based intervention (SBI) group or a telerehabilitation sensory-based intervention (TBSI) group. Both groups received weekly face-to-face sensory-based therapy for eight weeks. Additionally, the TBSI group participated in 30-minute weekly home-based telerehabilitation sessions. Outcome measures included the Canadian Occupational Performance Measure (COPM), the Functional Independence Measure for Children (WeeFIM), and the Dunn Sensory Profile.

**Results:**

Both groups demonstrated statistically significant improvements; however, the TBSI group showed greater gains in WeeFIM motor, cognitive, and total scores as well as COPM performance and satisfaction scores (p < 0.01). Furthermore, larger improvements and greater effect sizes were observed in the sensory processing subdomains of the TBSI group. Parental training and active participation appeared to enhance the effectiveness of the telerehabilitation program.

**Conclusions:**

Telerehabilitation is an effective intervention for improving motor and cognitive functions, sensory processing, and daily life participation in children with DCD. The findings support the integration of telerehabilitation into sensory-based approaches as part of a holistic model of care in occupational therapy practice.

**Trial registration:**

Clinicaltrials.gov NCT06977256.

## Introduction

Developmental Coordination Disorder (DCD) is a neurodevelopmental condition characterized by age-inappropriate development of motor skills. Difficulties in motor learning and problems with coordination. Children with DCD experience challenges in performing motor tasks that their typically developing peers can easily accomplish. These difficulties manifest as significant limitations in daily activities such as schoolwork, household tasks, leisure, and play [1,2]. DCD affects not only motor functions but also the individual’s psychosocial development. Children with DCD are frequently reported to experience emotional and social problems, including anxiety, low self-esteem, worry, and depression [3]. The disorder refers to children who, despite having a typical level of intelligence and no identifiable medical condition, demonstrate notable deficits in motor skills. Commonly observed symptoms include impairments in fine and gross motor coordination. Speech difficulties, abnormal muscle tone, and poor body awareness. These deficits result in substantial challenges in motor planning. Organizing movements and adapting motor responses when task demands change [4].

Children with DCD often encounter functional difficulties at home (e.g., dressing, eating), at school (e.g., handwriting, organizational skills), and in community settings (e.g., riding a bicycle, drawing, or participating in physical and recreational activities). These challenges may negatively impact academic achievement and social participation, leading to a decline in self-efficacy and quality of life in the long term [5]. Interventions targeting DCD should aim to promote independence and success in daily living activities. Structured and repetitive practices are essential to help children learn specific tasks. These interventions must also include the education and involvement of parents and teachers, as their support plays a critical role in reducing environmental barriers and enhancing the child’s functional performance [6].

Exercise and sensory-based interventions are designed to promote the child’s active engagement while also encouraging environmental adaptations and involving families in the therapeutic process. The literature emphasizes that individualized strategies addressing performance difficulties can meaningfully improve quality of life. The motor impairments and associated limitations in activity participation seen in children with DCD highlight the importance of early intervention. Therapeutic approaches aimed at facilitating active participation in everyday life support not only developmental progress but also help prevent long-term difficulties [7,8].

In recent years, telerehabilitation has emerged as an effective method for remotely managing patient care. Through these technologies, clinicians can evaluate patients and deliver individualized treatment guidance. Telerehabilitation is particularly valuable for reducing healthcare disparities in rural and underserved areas. Although studies remain limited, research has shown that video-supported home exercise programs delivered through web-based platforms can produce clinically meaningful outcomes. Current findings in the field of rehabilitation suggest that these programs can yield results comparable to conventional face-to-face treatment [9–11]. A recent meta-analysis demonstrated the efficacy of real-time telerehabilitation for musculoskeletal conditions. Various tools, such as telephone and video conferencing applications, have been shown to effectively deliver therapeutic interventions remotely [12]. Telerehabilitation is recognized as a comprehensive rehabilitation model encompassing services such as monitoring, intervention, consultation, education, and evaluation [13,14]. The literature shows that telerehabilitation improves accessibility, enhances treatment adherence, and increases patient satisfaction [11,15,16]. For children in particular, gamified virtual platforms have been found to improve motivation and participation in therapy. These programs also offer personalized content tailored to each child’s developmental pace and needs, enabling a flexible and adaptive intervention process. Home-based applications facilitate parental involvement and allow children to engage in therapy within a comfortable and familiar environment [17].

In this context. the present study aims to investigate the effects of a telerehabilitation-based intervention on motor performance, activity participation, and daily functioning in children within the developmental coordination disorder spectrum. Our hypothesis is that telerehabilitation, when provided in addition to sensory-based interventions, will lead to greater improvements in motor performance, activity engagement, and sensory abilities compared to sensory-based intervention alone.

## Methods

### Study design

This single-blind (participant), parallel-group randomized controlled trial with a 1:1 allocation ratio was conducted at the Gunisigi Counseling Center between 20/07/2023 and 5/12/2024. Ethical approval was granted by the Non-Interventional Clinical Research Ethics Committee of Istanbul Medipol University (Decision No: 587; Approval No: 10840098-772.02-4262; Date: July 13/07/2023). The study protocol and ethics approval documentation were meticulously developed and formally submitted to the institutional ethics committee prior to the commencement of any research procedures. The study protocol was subsequently registered on ClinicalTrials.gov (Identifier: NCT06977256). Although participant enrollment was initiated shortly before the formal completion of the ClinicalTrials.gov registration due to operational requirements and unforeseen scheduling constraints, the registration was finalized promptly upon recognition of this discrepancy. Notwithstanding this deviation, the research was carried out in strict adherence to internationally recognized ethical principles and clinical research regulations. The authors affirm that all current and associated clinical trials related to the intervention have been duly registered in accordance with applicable standards. As the participants were minors, both written and verbal informed consent were obtained from their legal guardians. The study adhered to the ethical principles of the Declaration of Helsinki and was reported in accordance with the CONSORT (Consolidated Standards of Reporting Trials) guidelines.

### Participants

This study was conducted with children aged between 3 and 7 years who were within the spectrum of DCD. The DCD diagnoses of all participants were confirmed by either a pediatric neurologist or a child psychiatrist. Children included in the study were required to be able to follow verbal instructions and participate in structured activities with caregiver support. Informed consent was obtained from the legal guardians of all participants. The inclusion criteria were as follows: (1) being between 3 and 7 years of age. (2) having a confirmed diagnosis of DCD by a pediatric neurologist or child psychiatrist. (3) having the cognitive capacity to participate actively in the sessions. (4) having received occupational therapy for a duration between 3 months and 1 year to ensure group homogeneity; and (5) obtaining written informed consent from the caregiver. The exclusion criteria were as follows: (1) having a diagnosis of moderate to severe intellectual disability or an autism spectrum disorder not associated with DCD. (2) having an uncorrected visual or hearing impairment. (3) having a systemic neurological or an orthopedic condition that may affect motor function, and (4) currently receiving occupational therapy or a similar rehabilitation intervention from another institution.

**Fig 1:**
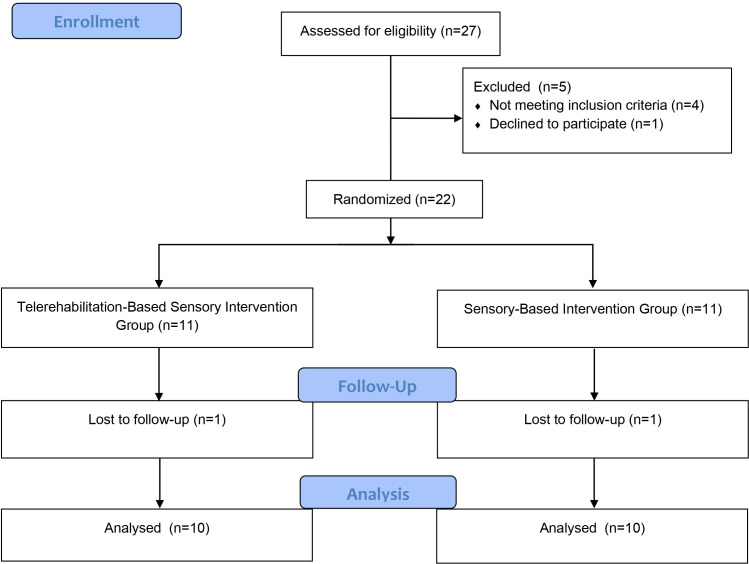
Design and flow chart of the study.

A total of 27 children diagnosed with DCD were initially screened for participation in the study. Four children were excluded for not meeting the inclusion criteria, and one child was excluded due to refusal to participate. As a result. Twenty-two participants were randomly assigned into two equal groups (n = 11 each). Both groups began the intervention phase with 11 participants. During the course of the intervention, one participant from each group withdrew due to illness and was excluded from further analysis. Consequently, study was completed with 10 participants in each group (Fig 1).

### Randomization and blinding

Randomization was carried out by an independent researcher who was not involved in any stage of the study, including participant recruitment. For assessment or intervention delivery, numbers from 1 to 22 were randomly assigned to two groups using the randomizer.org website. Participants were given a number according to their order of admission to the clinic. Based on these assigned numbers, the first 11 participants were allocated to the Sensory-Based Intervention (SBI) Group, and the remaining 11 participants to the Telerehabilitation-Based Sensory Intervention (TBSI) Group. To minimize the risk of bias, participants were blinded to their group allocation and were not informed about the content or format of the interventions provided to the other group. Due to the nature of the intervention protocols, blinding of outcome assessors was not possible.

## Interventions

### Sensory-based intervention group

**Fig 2:**
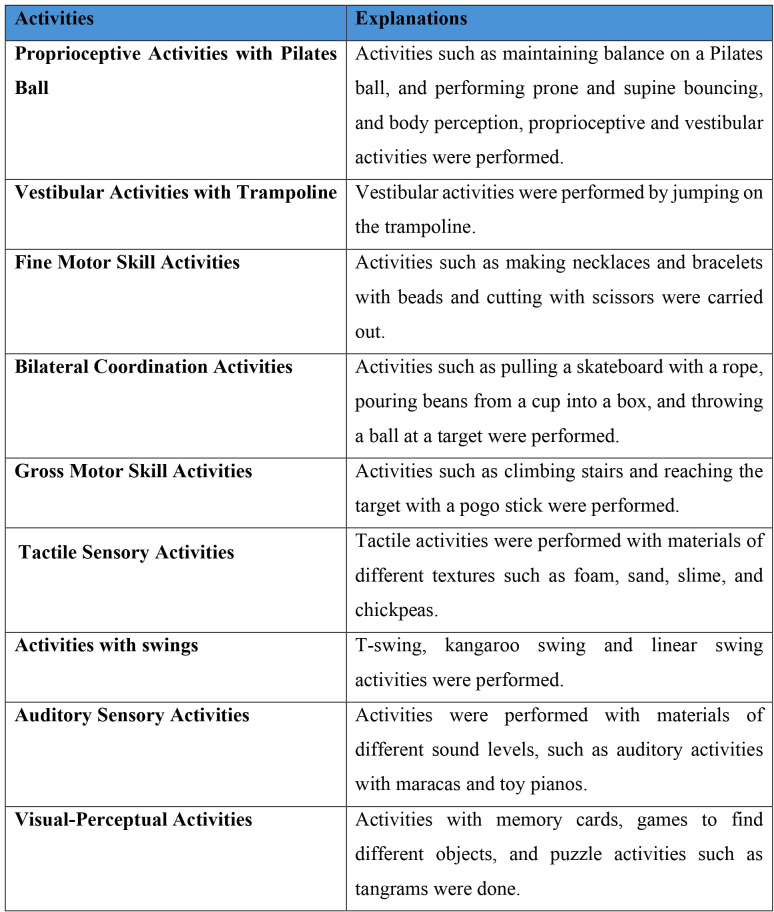
Structured sensory-based activities implemented in the SBI group.

The SBI Group received structured sensory-based therapy sessions face-to-face once a week for 40 minutes over 8 weeks. Participants attended these sessions in a therapy room equipped with sensory integration materials. The intervention program included a range of activities targeting proprioceptive, vestibular, tactile, auditory, and visual sensory domains. Example activities included balancing and bouncing on a pilates ball, jumping on a trampoline, linear swinging using various types of swings, and sensory play with materials such as foam and sand. Fine motor tasks such as bead stringing and cutting with scissors, and coordination exercises such as object transfer and ball targeting. Additionally, visual and auditory stimuli were incorporated through memory card games and musical toys. Parents were informed about the session content at the end of each session. Their questions were addressed, and personalized home assignments were provided. The content and difficulty level of the activities were individualized and planned according to each child’s developmental level and functional performance (Figure 2).

### Telerehabilitation-based sensory intervention group

Participants in the TBSI Group initially received face-to-face structured sensory-based therapy sessions once a week for 40 minutes over a period of 8 weeks. This was followed by an additional 8-week period during which participants received 30-minute telerehabilitation sessions once per week. The telerehabilitation sessions were delivered via the Zoom platform using a computer and webcam and served as a continuation of the previous in-person intervention to ensure treatment continuity. During the telerehabilitation phase, participants were provided with a variety of individualized supports, including remote parental guidance, ergonomic and environmental modifications, home-based activity recommendations, and structured caregiver instructions. Ergonomic adjustments were made according to each child’s motor needs and comfort, such as rearranging furniture and minimizing environmental distractions. Toys and materials were adapted to meet developmental needs, ensuring both the quality and quantity of sensory input. Individualized home programs were assigned, which included activities such as bouncing on a Pilates ball, swinging in a kangaroo-style swing, and tactile play with foam. Remote parental guidance was implemented to support sensory and motor development in daily routines by providing caregivers with step-by-step instructions during the sessions. Before each session, caregivers were informed about the necessary materials, appropriate toy selection, and optimal camera placement. All sessions were individualized, and activity difficulty levels were gradually increased from simple to complex based on each child’s functional capacity. Throughout the intervention, a stable virtual environment was maintained to allow uninterrupted visual and auditory interaction between the therapist and participant.

## Outcome measures

Outcome measures were administered to all participants both prior to the initiation of the intervention and following the completion of the eight-week treatment period.

### Primary outcomes

#### The Canadian occupational performance measure.

The Canadian Occupational Performance Measure (COPM) is a client-centered outcome tool specifically designed for occupational therapists to evaluate performance in the domains of self-care, productivity, and leisure. It utilizes a semi-structured interview format to facilitate individualized assessment. During the interview, participants identify activities they find problematic and subsequently rate their performance and satisfaction on a 10-point Likert scale, where 1 indicates the lowest and 10 the highest level. This process yields two distinct scores: one for perceived performance and the other for satisfaction with performance [18].

#### Dunn sensory profile parent questionnaire.

In this study, the Dunn Sensory Profile Parent Questionnaire was administered to evaluate the sensory processing profiles of participating children. Developed by Winnie Dunn in 1999 this standardized assessment tool is designed for children aged 3–10 years and consists of 125 items. The measure is structured around four primary patterns of sensory responsiveness: sensitivity, registration, avoidance, and seeking. Scoring is organized into three major domains: sensory processing, modulation, behavioral, and emotional responses. Subsections include auditory, visual, vestibular, tactile, multisensory, and oral sensory processing, as well as functional components such as posture, motor planning, activity level, and emotional regulation [19].

### Secondary outcome

#### Functional Independence Measure (WeeFIM).

The WeeFIM was developed to assess the motor and cognitive components of individuals’ functional abilities. It was designed to provide a unified disability assessment system based on the International Classification of Impairments, Disabilities, and Handicaps, tailored for use within the medical system in the United States. The WeeFIM is divided into two main domains: Motor and Cognitive. It consists of a total of 18 items, including 13 items in the motor domain and five items in the cognitive domain [20].

### Statistical analysis

An a priori power analysis was conducted using G*Power (version 3.1.9.7) to determine the minimum sample size required to detect statistically significant differences in both within-group and between-group comparisons. A previous randomized controlled trial targeting children with Developmental Coordination Disorder (Wood et al., 2017) reported large effect sizes (Cohen’s d ≈ 1.2–1.6) for motor and sensorimotor outcomes [21]. Based on these findings, we conservatively assumed a large effect size (d ≈ 0.80) for our primary outcomes. Assuming a power (1–β) of 0.80 and a significance level (α) of 0.05 for two-tailed tests, the required sample size was calculated to be 18 participants (n = 9 per group). To account for potential dropouts, we initially enrolled 22 participants (11 per group). During the intervention, one participant from each group withdrew, resulting in a final analyzed sample of 20 participants (n = 10 per group). This sample size was deemed sufficient to detect clinically meaningful differences using the planned statistical methods.

Statistical analyses were conducted using the Statistical Package for the Social Sciences (SPSS) version 25.0. The Shapiro-Wilk test was used to assess the normality of the data distribution. Descriptive statistics were reported as mean (M), standard deviation (SD)A significance level of p < 0.05 was considered statistically significant for all analyses. For between-group comparisons, the Independent Samples t-test was applied for normally distributed data, while the Mann–Whitney U test was used for non-normally distributed variables. For within-group comparisons, the Paired Samples t-test was used for normally distributed variables, whereas the Wilcoxon Signed-Rank test was applied to non-normally distributed variables. Cohen’s d was calculated for both within- and between-group comparisons and interpreted as follows: small (0.20–0.49), medium (0.50–0.79), and large (≥ 0.80) [22].

## Results

At baseline, the TBSI and SBI groups differed significantly on several measures. The TBSI group demonstrated higher COPM performance scores (p = 0.009) and lower COPM satisfaction scores (p = 0.004) compared to the SBI group. Significant differences were also observed in several sensory processing domains, including Sensitivity (p = 0.001), Avoiding (p < 0.001), Low Endurance/Tone (p = 0.022), and Oral Sensory Sensitivity (p = 0.003). No other significant differences were found between groups at baseline (p > 0.05) (Table 1).

**Table 1:** Comparison of pre-treatment measurements between groups.

Variables	TBSI Group (Mean ± SD)	SBI Group (Mean ± SD)	p
**WeeFIM Motor Domain**	55.70 ± 13.61	54.50 ± 10.40	0.970^a^
**WeeFIM/Cognitive Domain**	15.90 ± 8.02	14.30 ± 5.85	0.791^b^
**WeeFim Total**	71.60 ± 20.85	68.80 ± 15.83	0.850^b^
**COPM Performance**	34.30 ± 3.97	32.30 ± 3.68	0.009^b^
**COPM Satisfaction**	12.20 ± 3.77	17.9 ± 3.07	0.004^b^
**Registration**	34.80 ± 6.33	32.50 ± 3.74	0.518^a^
**Seeking**	83.20 ± 8.87	86.0 ± 9.58	0.271^a^
**Sensitivity**	56.90 ± 9.46	41.60 ± 5.12	0.001^a^
**Avoiding**	68.60 ± 8.43	49.0 ± 7.55	0.001^a^
**Sensation seeking**	53.0 ± 6.42	56.90 ± 7.88	0.138^a^
**Emotional reactive**	19.60 ± 4.50	18.40 ± 2.50	0.970^a^
**Low endurance/tone**	21.50 ± 4.11	16.80 ± 4.02	0.022^a^
**Oral sensory sensitivity**	29.80 ± 7.05	18.40 ± 4.30	0.003^a^
**Inattention/distractibility**	16.30 ± 4.16	14.40 ± 1.77	0.444^b^
**Poor registration**	20. 90 ± 4.22	19.0 ± 2.49	0.271^b^
**Sensory sensitivity**	10.40 ± 1.95	8.80 ± 2.04	0.071^b^
**Sedentary**	16.40 ± 2.31	8.60 ± 2.71	<.001^b^
**Fine motor/perceptual**	6.80 ± 1.68	5.50 ± 1.17	0.126^b^
**Auditory processing**	19.70 ± 6.46	15.90 ± 2.47	0.145^b^
**Visual processing**	23.60 ± 5.40	17.90 ± 2.37	0.013^b^
**Vestibular processing**	26.90 ± 4.01	32.70 ± 4.59	0.006^b^
**Touch processing**	52.10 ± 5.25	47.70 ± 3.94	0.087^b^
**Multisensory processing**	16.10 ± 3.92	18.30 ± 3.19	0.224^b^
**Oral sensory processing**	38.70 ± 9.45	28.30 ± 4.13	0.023^b^
**Sensory processing related to endurance/tone**	21.50 ± 4.11	16.80 ± 4.02	0.022^a^
**Modulation related to body position and movement**	34.10 ± 5.78	30.50 ± 4.06	0.034^b^
**Modulation of movement affecting activity level**	24.50 ± 1.95	15.20 ± 3.82	<.001^b^
**Modulation of sensory input affecting emotional responses and activity level**	9.30 ± 1.41	7.60 ± 1.83	0.069^a^
**Modulation of visual input affecting emotional responses and activity level**	11.90 ± 1.81	9.70 ± 2.05	0.021^a^
**Emotional/social responses**	22.10 ± 6.04	18.80 ± 2.82	0.126^a^
**Behavioral outcomes of sensory processing**	25.10 ± 5.30	22.80 ± 3.73	0.383^a^
**Items indicating thresholds for response**	8.10 ± 1.28	7. 30 ± 1.88	0.604^a^

TBSI: Telerehabilitation-Based Sensory Intervention; SBI: Sensory-Based Intervention; COPM: Canadian Occupational Performance Measure.^a^Mann–Whitney U test,^b^Independent Samples t-test.

Following the intervention, the TBSI group exhibited statistically significant improvements across nearly all outcome measures. Functional performance increased substantially: WeeFIM Motor (p = 0.002, d = 1.21), Cognitive (p = 0.006, d = 1.53), and Total scores (p = 0.006, d = 4.22) all showed large to very large effect sizes. COPM performance and satisfaction scores also improved markedly (p = 0.006 for both), with effect sizes of d = 4.29 and d = 5.71, respectively. Sensory processing outcomes improved significantly in the TBSI group, with large effects observed across most subscales. For instance, Registration (p = 0.006, d = 4.82), Sensitivity (p = 0.002, d = 3.49), Low Endurance/Tone (p = 0.006, d = 4.88), and Emotional Reactivity (p = 0.006, d = 3.29) demonstrated very large improvements. Even Oral Sensory Sensitivity, which showed a smaller change, reached statistical significance (p = 0.036, d = 0.74). The only outcome that did not improve significantly was the Sedentary subscale (p = 0.265, d = –0.43) (Table 2). In the SBI group, significant pre–post improvements were also observed. Moderate effect sizes were found for WeeFIM Motor (p = 0.006, d = 0.68), Cognitive (p = 0.006, d = 0.59), and Total scores (p = 0.006, d = 0.68). COPM performance and satisfaction scores demonstrated large effects (p = 0.006 for both), with d = 3.34 and d = 4.25, respectively. Several sensory domains also improved significantly in the SBI group, including Registration (p = 0.008, d = 1.02), Sensitivity (p = 0.006, d = 1.68), Avoiding (p = 0.006, d = 1.85), and Emotional Reactivity (p = 0.006, d = 1.58), all with large effect sizes. The Sedentary subscale improved moderately (p = 0.020, d = 0.82), and several other subscales demonstrated effect sizes between d ≈ 1.1 and 1.9. However, some outcomes in the SBI group did not reach statistical significance. Specifically, Seeking (p = 0.539, d = 0.27), Sensation Seeking (p = 0.798, d = 0.23), Vestibular Processing (p = 0.181, d = 0.43), and Modulation related to Body Position (p = 1.000, d = 0.26) showed negligible effects. Other subscales that significantly improved generally demonstrated moderate to large effects (d ≈ 0.6–1.2). Non-significant outcomes consistently yielded small effect sizes (d < 0.30) (Table 2).

**Table 2:** Comparison of changes in outcome measures within and between the groups.

Variables	Pre-TBSI Group (Mean ± SD)	Post-TBSI Group (Mean ± SD)	Cohen d	p	Pre-SBI Group (Mean±SD)	Post-SBI Group (Mean±SD)	Cohen d	p
**WeeFIM Motor Domain**	55.70 ± 13.61	70.70 ± 11.04	1.21	0.002^a^	54.50 ± 10.40	61.00 ± 8.61	0.68	0.006^a^
**WeeFIM/Cognitive Domain**	15.90 ± 8.02	27 ± 6.36	1.53	0.006^b^	14.30 ± 5.85	17.80 ± 6.03	0.59	0.006^b^
**WeeFim Total**	71.60 ± 0.85	91.70 ± 6.68	4.22	0.006^b^	68.80 ± 15.83	78.80 ± 13.54	0.68	0.006^b^
**COPM Performance**	12.10 ± 3.25	30.0 ± 4.92	4.29	0.006^b^	16.2 ± 3.12	27.4 ± 3.57	3.34	0.006^b^
**COPM Satisfaction**	12.20 ± 3.77	34.3 ± 3.97	5.71	0.006^b^	17.9 ± 3.07	32.3 ± 3.68	4.25	0.006^b^
**Registration**	34.80 ± 6.33	59.0 ± 3.23	4.82	0.006^a^	32.50 ± 3.74	38.30 ± 7.13	1.02	0.008^a^
**Seeking**	83.20 ± 8.87	102.50 ± 8.01	2.28	0.006^a^	86.0 ± 9.58	88.10 ± 5.34	0.27	0.539^a^
**Sensitivity**	56.90 ± 9.46	83.90 ± 5.50	3.49	0.002^a^	41.60 ± 5.12	55.60 ± 10.59	1.68	0.006^a^
**Avoiding**	68.60 ± 8.43	99.50 ± 7.44	3.89	0.006^a^	49.0 ± 7.55	62.90 ± 7.46	1.85	0.006^a^
**Sensation seeking**	53.0 ± 6.42	67.20 ± 4.94	2.48	0.006^a^	56.90 ± 7.88	58.30 ± 3.40	0.23	0.798^a^
**Emotional reactive**	19.60 ± 4.50	34.50 ± 4.55	3.29	0.006^a^	18.40 ± 2.50	23.0 ± 3.26	1.58	0.006^a^
**Low endurance/tone**	21.50 ± 4.11	35.90 ± 0.73	4.88	0.006^a^	16.80 ± 4.02	22.20 ± 5.02	1.19	0.006^a^
**Oral sensory sensitivity**	29.80 ± 7.05	34.10 ± 4.22	0.74	0.036^a^	18.40 ± 4.30	23.60 ± 6.56	0.94	0.005^a^
**Inattention/distractibility**	16.30 ± 4.16	28.10 ± 2.37	3.49	0.006^b^	14.40 ± 1.77	19.0 ± 2.90	1.91	0.006^b^
**Poor registration**	20.90 ± 4.22	31.60 ± 2.54	3.07	0.006^b^	19.0 ± 2.49	22.0 ± 1.49	1.46	0.016^b^
**Sensory sensitivity**	10.40 ± 1.95	15.60 ± 0.96	3.38	0.005^b^	8.80 ± 2.04	10.50 ± 2.06	0.83	0.021^b^
**Sedentary**	16.40 ± 2.31	15.60 ± 1.26	−0.43	0.265^b^	8.60 ± 2.71	10.40 ± 1.50	0.82	0.020^b^
**Fine motor/perceptual**	6.80 ± 1.68	11.60 ± 0.96	3.51	0.005^b^	5.50 ± 1.17	7.20 ± 1.47	1.28	0.014^b^
**Auditory processing**	19.70 ± 6.46	32.20 ± 2.93	2.49	0.006^b^	15.90 ± 2.47	21.40 ± 3.53	1.81	0.006^b^
**Visual processing**	23.60 ± 5.40	33.30 ± 3.65	2.10	0.006^b^	17.90 ± 2.37	22.70 ± 3.05	1.76	0.006^b^
**Vestibular processing**	26.90 ± 4.01	44.50 ± 2.95	5.00	0.006^b^	32.70 ± 4.59	34.30 ± 2.66	0.43	0.181^b^
**Touch processing**	52.10 ± 5.25	72.50 ± 6.04	3.61	0.006^b^	47.70 ± 3.94	54.70 ± 3.49	1.88	0.006^b^
**Multisensory processing**	16.10 ± 3.92	27.10 ± 2.92	3.18	0.006^b^	18.30 ± 3.19	20.30 ± 3.36	0.61	0.029^b^
**Oral sensory processing**	38.70 ± 9.45	46.0 ± 5.33	0.95	0.006^b^	28.30 ± 4.13	33.40 ± 7.27	0.86	0.005^b^
**Sensory processing related to endurance/tone**	21.50 ± 4.11	35.90 ± 0.73	4.88	0.006^a^	16.80 ± 4.02	22.20 ± 5.02	1.19	0.006^a^
**Modulation related to body position and movement**	34.10 ± 5.78	39.20 ± 1.68	1.20	0.038^b^	30.50 ± 4.06	31.40 ± 2.71	0.26	1.000^b^
**Modulation of movement affecting activity level**	24.50 ± 1.95	27.30 ± 1.49	1.61	0.009^b^	15.20 ± 3.82	18.60 ± 2.11	1.10	0.009^b^
**Modulation of sensory input affecting emotional responses and activity level**	9.30 ± 1.41	15.30 ± 1.49	4.14	0.006^a^	7.60 ± 1.83	9.60 ± 1.83	1.09	0.021^a^
**Modulation of visual input affecting emotional responses and activity level**	11.90 ± 1.81	16.0 ± 1.24	2.64	0.006^a^	9.70 ± 2.05	11.30 ± 1.41	0.91	0.020^a^
**Emotional/social responses**	22.10 ± 6.04	38.50 ± 4.69	3.03	0.006^a^	18.80 ± 2.82	24.40 ± 3.20	1.86	0.006^a^
**Behavioral outcomes of sensory processing**	25.10 ± 5.30	43.10 ± 4.63	3.62	0.006^a^	22.80 ± 3.73	28.60 ± 3.23	1.66	0.008^a^
**Items indicating thresholds for response**	8.10 ± 1.28	11.50 ± 1.08	2.87	0.005^a^	7.30 ± 1.889	9.20 ± 1.619	1.08	0.019^a^

TBSI: Telerehabilitation-Based Sensory Intervention; SBI: Sensory-Based Intervention; COPM: Canadian Occupational Performance Measure. ^a^Wilcoxon Signed-Rank test,^b^Paired Samples t-test.

Between-group comparisons revealed significantly greater improvements in the TBSI group across most outcomes. Of the 32 variables analyzed, 26 showed statistically significant between-group differences (p < 0.05), with Cohen’s d ranging from −1.40 to −4.07. The largest difference was found in Vestibular Processing (p < 0.001, d = –4.07). Other domains with large effect sizes favoring the TBSI group included Registration (p < 0.001, d = –2.91), Multisensory Processing (p < 0.001, d = –3.12), and Touch Processing (p < 0.001, d = –2.42).

Functional outcomes also favored the TBSI group, with significant between-group differences in WeeFIM Motor (p = 0.015, d = –1.44), Cognitive (p < 0.001, d = –2.17), and Total scores (p = 0.003, d = –1.80). COPM Satisfaction was significantly higher in the TBSI group (p = 0.004, d = 1.66).In contrast, six outcomes did not differ significantly between groups: COPM Performance (p = 0.254, d = 1.29), Oral Sensory Sensitivity (p = 0.442, d = 0.20), Oral Sensory Processing (p = 0.701, d = –0.37), Modulation Related to Body Position and Movement (p = 0.185, d = –0.69), Modulation of Movement Affecting Activity Level (p = 0.564, d = 0.30), and Items Indicating Thresholds for Response (p = 0.055, d = –0.92) (Table 3).

**Table 3:** Differences within groups before and after treatment and comparison of differences between groups.

Variables	TBSI Group (Mean ± SD)	SBI Group (Mean ± SD)	Cohen d	p
**WeeFIM Motor Domain**	15.00 ± 7.69	6.50 ± 3.27	−1.44	0.015^a^
**WeeFIM/Cognitive Domain**	11.10 ± 4.72	3.50 ± 1.51	−2.17	<.001^b^
**WeeFim Total**	26.100 ± 12.20	10.00 ± 3.33	−1.80	0.003^b^
**COPM Performance**	12.10 ± 3.25	16.2 ± 3.12	1.29	0.254^b^
**COPM Satisfaction**	12.20 ± 3.77	17.9 ± 3.07	1.66	0.004^b^
**Registration**	24.20 ± 7.22	5.80 ± 5.29	−2.91	<.001^a^
**Seeking**	19.30 ± 10.26	2.10 ± 10.46	−1.66	0.004^a^
**Sensitivity**	27.00 ± 6.36	14.00 ± 7.59	−1.86	0.002^a^
**Avoiding**	30.90 ± 9.56	13.90 ± 4.77	−2.25	<.001^a^
**Sensation seeking**	14.20 ± 8.16	1.40 ± 7.38	−1.65	0.004^a^
**Emotional reactive**	14.90 ± 5.36	4.60 ± 2.41	−2.48	<.001^a^
**Low endurance/tone**	14.40 ± 4.38	5.40 ± 2.59	−2.50	<.001^a^
**Oral sensory sensitivity**	4.30 ± 5.56	5.20 ± 3.12	0.20	0.442^a^
**Inattention/distractibility**	11.80 ± 3.65	4.60 ± 2.95	−2.17	<.001^b^
**Poor registration**	10.70 ± 4.03	3.0 ± 2.75	−2.23	<.001^b^
**Sensory sensitivity**	5.20 ± 2.10	1.70 ± 1.49	−1.92	0.001^b^
**Sedentary**	−0.80 ± 1.75	1.80 ± 1.40	1.64	0.003^b^
**Fine motor/perceptual**	4.80 ± 1.48	1.70 ± 1.34	−2.20	0.001^b^
**Auditory processing**	12.50 ± 5.50	5.50 ± 3.69	−1.49	0.004^b^
**Visual processing**	9.70 ± 4.24	4.80 ± 2.53	−1.40	0.009^b^
**Vestibular processing**	17.60 ± 4.27	1.60 ± 3.57	−4.07	<.001^b^
**Touch processing**	20.40 ± 6.10	7.0 ± 4.90	−2.42	<.001^b^
**Multisensory processing**	11.00 ± 3.33	2.0 ± 2.36	−3.12	<.001^b^
**Oral sensory processing**	7.30 ± 7.09	5.10 ± 4.46	−0.37	0.701^b^
**Sensory processing related to endurance/tone**	14.40 ± 4.38	5.40 ± 2.59	−2.50	<.001^a^
**Modulation related to body position and** **movement**	5.10 ± 6.71	0.90 ± 5.30	−0.69	0.185^b^
**Modulation of movement affecting activity level**	2.80 ± 1.75	3.40 ± 2.22	0.30	0.564^b^
**Modulation of sensory input affecting emotional responses and activity level**	6.00 ± 2.11	2.0 ± 1.63	−2.12	0.001^a^
**Modulation of visual input affecting emotional responses and activity level**	4.10 ± 1.66	1.60 ± 1.51	−1.58	0.004^a^
**Emotional/social responses**	16.40 ± 6.45	5.60 ± 2.17	−2.24	<.001^a^
**Behavioral outcomes of sensory processing**	18.0 ± 5.89	5.80 ± 4.05	−2.41	<.001^a^
**Items indicating thresholds for response**	3.40 ± 1.51	1.90 ± 1.73	−0.92	0.055^a^

TBSI: Telerehabilitation-Based Sensory Intervention; SBI: Sensory-Based Intervention; COPM: Canadian Occupational Performance Measure.^a^Mann–Whitney U test,^b^Independent Samples t-test.

In summary, the TBSI group exhibited superior therapeutic gains across a wide range of sensory, functional, and behavioral outcomes compared to the SBI group, particularly in sensory modulation and integration domains.

## Discussion

This study examined the effects of telerehabilitation on activity performance and participation in children within the DCD spectrum. Our findings demonstrated that telerehabilitation led to significant improvements, particularly in motor and cognitive domains. In the TBSI group, significant increases were observed in pre- and post-treatment WeeFIM Motor, Cognitive, and Total scores, suggesting that telerehabilitation may effectively enhance functional independence in this population. Furthermore, improvements in COPM performance and satisfaction scores indicated that telerehabilitation positively impacts not only skill development but also participation and perceived competence.

DCD is known to adversely affect motor performance, academic achievement, social skills, and psychological well-being. Biotteau et al. emphasized the importance of individualized intervention planning and suggested that a multidisciplinary and holistic approach may enhance quality of life in children with DCD [23]. Numerous studies have shown that sensory-based interventions, widely used in the treatment of DCD, positively influence motor coordination and participation in daily activities [7]. In line with this, both groups in our study received a common sensory-based intervention to ensure methodological consistency and to evaluate the general effects of sensory integration-based therapy. The findings revealed significant improvements in both groups, suggesting that such approaches contribute meaningfully to functional outcomes in children.

Telerehabilitation has been reported as a safe and effective alternative in situations where face-to-face therapy access is limited [24]. In children with autism spectrum disorder (ASD), telerehabilitation has been shown to yield significant improvements in motor skills [25]. Wang et al. further suggested that tailoring telerehabilitation programs to individual needs may enhance their effectiveness [26]. A systematic review found that web-based telerehabilitation significantly improves motor function and physical activity levels in children, while also facilitating access to care for families living in remote areas [26]. The significant improvements in WeeFIM scores in our study are consistent with these findings and support the utility of telerehabilitation as an effective intervention model. Gains in sensory processing parameters further reinforce the improvements observed in motor performance.

In our study, parents in the TBSI group received training on home-based therapeutic activities and were encouraged to participate actively in the rehabilitation process. The involvement of caregivers appeared to play a critical role in improving activity performance, participation, motor, and sensory skills. Parental engagement enabled therapy to be implemented in the child’s natural environment, thereby promoting smoother integration into daily life. Morriss Tkach et al. reported that parents assumed multiple roles such as observer, assistant, or implementer during telerehabilitation sessions, which facilitated the child’s engagement in therapy [27]. Similarly, McQueen et al. evaluated a parent-mediated physical activity program delivered via a Facebook group and found that 78% of caregivers followed the content and over 80% reported the program to be beneficial [28]. These studies support the idea that parent education interventions not only increase parental self-efficacy but also positively influence children’s developmental outcomes. Active parental involvement is recognized as a fundamental component for the success of pediatric telerehabilitation. Parents play a critical role in encouraging their child’s engagement during therapy, ensuring consistent participation in sessions, and supporting therapeutic activities within the home environment, all of which facilitate the transfer of therapeutic gains into daily life [29,30]. However, parental involvement may be limited by several challenges. Difficulties such as time management, lack of digital literacy, technological infrastructure issues, and the child’s reduced ability to maintain attention during screen-based sessions can negatively affect the rehabilitation process. Socioeconomic disadvantages, in particular, may hinder some families’ access to and continuity in telerehabilitation programs. Therefore, when designing telerehabilitation interventions, it is crucial to provide families with technical support, flexible scheduling, and clear guidance. Active, informed, and confident parental participation significantly supports the child’s functional development and achievement of therapeutic goals [31–33].

The effectiveness of telerehabilitation in enhancing functional outcomes is also supported by a growing body of literature. A study conducted during the COVID-19 pandemic evaluated the efficacy of telerehabilitation using the COPM and found that it significantly contributed to achieving functional goals and improving performance and satisfaction in children with various diagnoses [34]. Kheirollahzadeh et al. reported that both telerehabilitation and face-to-face therapy positively impacted academic self-efficacy and activity performance in children with specific learning disabilities, with no significant difference between the two modalities [35].

Farahani et al. highlighted significant gains in performance and satisfaction scores following a Cognitive Orientation to daily Occupational Performance (CO-OP) intervention in children with autism and developmental delays [36], while James et al. reported similar positive findings with web-based telerehabilitation in children with cerebral palsy [37]. In our study, COPM-based evaluations revealed significant post-treatment improvements in performance and satisfaction, suggesting that telerehabilitation supports daily life integration and functional gains in children with DCD.

Our findings on sensory processing also support the efficacy of telerehabilitation. Significant improvements were observed in various sensory domains (e.g., movement/position, seeking, vestibular processing) in both groups. However, the TBSI group exhibited greater gains in most subdomains, as measured by the Dunn Sensory Profile. These results suggest that combining telerehabilitation with sensory integration-based interventions may enhance sensory functioning. Miller et al. similarly demonstrated in a randomized controlled trial that sensory integration-based occupational therapy may effectively alleviate sensory and behavioral challenges [38].

Furthermore, the notable improvements in motor and participation outcomes in the telerehabilitation group may reflect the benefits of delivering goal-oriented activities in children’s natural environments. Supporting this, Su et al. found that both telerehabilitation and face-to-face approaches were comparably effective in improving fine motor performance in children with ASD, and reported telerehabilitation as a feasible and efficient option [39].

The eight-week duration of intervention in our study is consistent with similar protocols in the literature, and our findings revealed that the TBSI group exhibited significantly greater improvements in motor outcomes compared to the SBI group. These findings suggest that increased parental involvement through telerehabilitation may help caregivers better understand their children’s motor needs and difficulties, thereby enhancing treatment outcomes. In conclusion, our results demonstrate that telerehabilitation is a promising intervention model that not only improves motor performance and participation but also enhances accessibility and parent satisfaction [26]. When combined with parent education, telerehabilitation contributes to both child development and family engagement in therapy. These findings indicate that telerehabilitation should be considered an integral component of a holistic care model for children within the DCD spectrum [30].

This study has several limitations. First, the relatively small sample size may limit the generalizability of the findings. Second, the follow-up period was restricted to eight weeks, which did not allow for the evaluation of long-term effects of the intervention. Additionally, the use of parent-reported outcome measures introduces the potential for subjective bias in the data. Therefore, the results of this study should be interpreted with caution and within the context of these methodological constraints.

## Conclusion

Telerehabilitation emerges as an effective intervention to improve motor performance, sensory processing, and daily activity participation in children with developmental coordination disorder. This study demonstrates that integrating telerehabilitation into sensory-based therapy may enhance functional outcomes, particularly when supported by active parental involvement. The findings advocate for the incorporation of telerehabilitation as a component of holistic pediatric rehabilitation models.

## Supporting information

S1 File CONSORT Checklist. (DOC)

## References

[1] Katartzi E, Kontou M, Pappas I, Trigonis I, Kourtessis T. Objective and subjective physical activity assessment in adolescents with motor difficulties. Children (Basel). 2025;12(4):488. doi: 10.3390/children12040488 40310162 PMC12025898

[2] Fujisawa S, Saito A, Sugawara M, Nakai A. Association between developmental coordination disorder traits, autistic traits, and emotional/behavioral problems in japanese preschool children. Children (Basel). 2025;12(4):420. doi: 10.3390/children12040420 40310059 PMC12025427

[3] Zwicker JG, Harris SR, Klassen AF. Quality of life domains affected in children with developmental coordination disorder: a systematic review. Child Care Health Dev. 2013;39(4):562–80. doi: 10.1111/j.1365-2214.2012.01379.x 22515477

[4] González López A, Crespo Madrid V, Hidalgo-Robles Á, Gutiérrez-Ortega M. Early signs of functioning and contextual factors in children 0 to 6 years of age at high risk of or with developmental coordination disorder: A scoping review. Child Care Health Dev. 2023;49: 230–9. doi: 10.1111/cch.13049 35998914

[5] de Oliveira Beliche TW, Weber MD, Tudella E, de Campos AC. Participation of Children With Developmental Coordination Disorder: A Scoping Review. Child Care Health Dev. 2025;51(2):e70059. doi: 10.1111/cch.70059 40090764

[6] Camden C, Wilson B, Kirby A, Sugden D, Missiuna C. Best practice principles for management of children with developmental coordination disorder (DCD): results of a scoping review. Child Care Health Dev. 2015;41(1):147–59. doi: 10.1111/cch.12128 24387638

[7] Yamanishi Y, Orita Y, Nagayoshi M, Nishimura R, Shinjyo T, Masuda K, et al. Examining the Effectiveness of Ayres Sensory Integration® Intervention for Children With Developmental Coordination Disorder in Improving Motor Coordination and Daily Activity Function: A Randomized Controlled Trial. Cureus. 2025;17(1):e76971. doi: 10.7759/cureus.76971 39917117 PMC11798754

[8] Alghadier M, Alhusayni AI. Evaluating the efficacy of gross-motor-based interventions for children with developmental coordination disorder: a systematic review. J Clin Med. 2024;13(16):4609. doi: 10.3390/jcm13164609 39200751 PMC11355478

[9] Tousignant M, Moffet H, Boissy P, Corriveau H, Cabana F, Marquis F. A randomized controlled trial of home telerehabilitation for post-knee arthroplasty. J Telemed Telecare. 2011;17(4):195–8. doi: 10.1258/jtt.2010.100602 21398389

[10] Hailey D, Roine R, Ohinmaa A, Dennett L. Evidence of benefit from telerehabilitation in routine care: a systematic review. J Telemed Telecare. 2011;17(6):281–7. doi: 10.1258/jtt.2011.101208 21844172

[11] Menek B, Dansuk E. Comparative Efficacy of Supervised, Web-Based, and Self-Guided Exercise Interventions in Women with Patellofemoral Pain Syndrome. Medicina. 2025;61(4):731. doi: 10.3390/medicina61040731 40283022 PMC12029018

[12] Cottrell MA, Galea OA, O’Leary SP, Hill AJ, Russell TG. Real-time telerehabilitation for the treatment of musculoskeletal conditions is effective and comparable to standard practice: a systematic review and meta-analysis. Clin Rehabil. 2017;31(5):625–38. doi: 10.1177/0269215516645148 27141087

[13] Gilbert AW, Jaggi A, May CR. What is the patient acceptability of real time 1:1 videoconferencing in an orthopaedics setting? A systematic review. Physiotherapy. 2018;104(2):178–86. doi: 10.1016/j.physio.2017.11.217 29361298

[14] Meijer HA, Graafland M, Goslings JC, Schijven MP. Systematic Review on the Effects of Serious Games and Wearable Technology Used in Rehabilitation of Patients With Traumatic Bone and Soft Tissue Injuries. Arch Phys Med Rehabil. 2018;99(9):1890–9. doi: 10.1016/j.apmr.2017.10.018 29138050

[15] Ramkumar PN, Haeberle HS, Ramanathan D, Cantrell WA, Navarro SM, Mont MA, et al. Remote Patient Monitoring Using Mobile Health for Total Knee Arthroplasty: Validation of a Wearable and Machine Learning-Based Surveillance Platform. J Arthroplasty. 2019;34(10):2253–9. doi: 10.1016/j.arth.2019.05.021 31128890

[16] Tripuraneni KR, Foran JRH, Munson NR, Racca NE, Carothers JT. A Smartwatch Paired With A Mobile Application Provides Postoperative Self-Directed Rehabilitation Without Compromising Total Knee Arthroplasty Outcomes: A Randomized Controlled Trial. J Arthroplasty. 2021;36(12):3888–93. doi: 10.1016/j.arth.2021.08.007 34462184

[17] Lino F, Arcangeli V, Chieffo DPR. The Virtual Challenge: Virtual Reality Tools for Intervention in Children with Developmental Coordination Disorder. Children (Basel). 2021;8(4):270. doi: 10.3390/children8040270 33915999 PMC8065642

[18] Law M, Baptiste S, McColl M, Opzoomer A, Polatajko H, Pollock N. The Canadian occupational performance measure: an outcome measure for occupational therapy. Can J Occup Ther. 1990;57(2):82–7. doi: 10.1177/000841749005700207 10104738

[19] Dunn W. Sensory Profile: User’s Manual. Psychological Corporation. 1999.

[20] Küçükdeveci AA, Yavuzer G, Elhan AH, Sonel B, Tennant A. Adaptation of the Functional Independence Measure for use in Turkey. Clin Rehabil. 2001;15(3):311–9. doi: 10.1191/026921501676877265 11386402

[21] Wood G, Miles CAL, Coyles G, Alizadehkhaiyat O, Vine SJ, Vickers JN, et al. A randomized controlled trial of a group-based gaze training intervention for children with Developmental Coordination Disorder. PLoS One. 2017;12(2):e0171782. doi: 10.1371/journal.pone.0171782 28187138 PMC5302797

[22] Cohen J. Statistical power analysis for the behavioral sciences. routledge; 2013. Available from: https://www.taylorfrancis.com/books/mono/10.4324/9780203771587/statistical-power-analysis-behavioral-sciences-jacob-cohen

[23] Biotteau M, Danna J, Baudou É, Puyjarinet F, Velay J-L, Albaret J-M, et al. Developmental coordination disorder and dysgraphia: signs and symptoms, diagnosis, and rehabilitation. Neuropsychiatr Dis Treat. 2019;15:1873–85. doi: 10.2147/NDT.S120514 31371960 PMC6626900

[24] Del Lucchese B, Parravicini S, Filogna S, Mangani G, Beani E, Di Lieto MC, et al. The wide world of technological telerehabilitation for pediatric neurologic and neurodevelopmental disorders - a systematic review. Front Public Health. 2024;12:1295273. doi: 10.3389/fpubh.2024.1295273 38694988 PMC11061864

[25] Aqdassi L, Sadeghi S, Pouretemad HR, Fathabadi J. A family-based telerehabilitation program for improving gross motor skills in children with high functioning autism spectrum disorder. J Mod Rehabil. 2021;15:173–82.

[26] Wang Z, He K, Sui X, Yi J, Yang Z, Wang K, et al. The Effect of Web-Based Telerehabilitation Programs on Children and Adolescents With Brain Injury: Systematic Review and Meta-Analysis. J Med Internet Res. 2023;25:e46957. doi: 10.2196/46957 38145485 PMC10775025

[27] Tkach MM, Earwood JH. Roles Caregivers Take on in Pediatric Rehabilitation Telehealth Services: A Scoping Review. OTJR (Thorofare N J). 2024. doi: 10.1177/15394492241291576 39498879

[28] McQueen M, Parker A, Pascoe M, Baldwin P, Mancini V, Cairney J, et al. Investigating the Feasibility and Acceptability of a Facebook Delivered, Parent Mediated, Physical Activity Intervention for Children with Developmental Coordination Disorder. International Journal of Disability, Development and Education. 2024:1–22. doi: 10.1080/1034912x.2024.2411265

[29] Camden C, Pratte G, Fallon F, Couture M, Berbari J, Tousignant M. Diversity of practices in telerehabilitation for children with disabilities and effective intervention characteristics: results from a systematic review. Disabil Rehabil. 2020;42(24):3424–36. doi: 10.1080/09638288.2019.1595750 30978110

[30] Ogourtsova T, Boychuck Z, O’Donnell M, Ahmed S, Osman G, Majnemer A. Telerehabilitation for Children and Youth with Developmental Disabilities and Their Families: A Systematic Review. Phys Occup Ther Pediatr. 2023;43(2):129–75. doi: 10.1080/01942638.2022.2106468 36042567

[31] Aarabi MA, Abdi K, Soleimani F. Tele-rehabilitation for children with physical disabilities: qualitative exploration of challenges in Iran. BMC Pediatr. 2025;25(1):11. doi: 10.1186/s12887-024-05341-6 39773713 PMC11705707

[32] Tully L, Case L, Arthurs N, Sorensen J, Marcin JP, O’Malley G. Barriers and Facilitators for Implementing Paediatric Telemedicine: Rapid Review of User Perspectives. Front Pediatr. 2021;9:630365. doi: 10.3389/fped.2021.630365 33816401 PMC8010687

[33] Philip J, Hussaindeen JR, Jacob N, Sethuraman S, Swaminathan M. Parental perception of facilitators and barriers to activity and participation in an integrated tele-rehabilitation model for children with cerebral visual impairment in South India - A virtual focus group discussion study. Indian J Ophthalmol. 2023;71(2):601–7. doi: 10.4103/ijo.IJO_1670_22 36727370 PMC10228909

[34] Tanner LR, Grinde K, McCormick C. The Canadian Occupational Performance Measure: a Feasible Multidisciplinary Outcome Measure for Pediatric Telerehabilitation. Int J Telerehabil. 2021;13(1):e6372. doi: 10.5195/ijt.2021.6372 34345346 PMC8287748

[35] Kheirollahzadeh M, Azad A, Saneii SH, Alizadeh Zarei M. Comparing Telerehabilitation and In-Person Interventions in School-Based Occupational Therapy for Specific Learning Disorder A Randomized Controlled Trial. Iran J Child Neurol. 2024;18(2):83–101. doi: 10.22037/ijcn.v18i2.43985 38617396 PMC11015722

[36] Kangarani-Farahani M, Thompson-Hodgetts S, Zwicker JG. Effectiveness of Cognitive Orientation to daily Occupational Performance for autistic children with developmental coordination disorder. Dev Med Child Neurol. 2025;67(2):216–22. doi: 10.1111/dmcn.16058 39141692 PMC11695746

[37] James S, Ziviani J, Ware RS, Boyd RN. Randomized controlled trial of web-based multimodal therapy for unilateral cerebral palsy to improve occupational performance. Dev Med Child Neurol. 2015;57(6):530–8. doi: 10.1111/dmcn.12705 25955443

[38] Miller LJ, Coll JR, Schoen SA. A randomized controlled pilot study of the effectiveness of occupational therapy for children with sensory modulation disorder. Am J Occup Ther. 2007;61(2):228–38. doi: 10.5014/ajot.61.2.228 17436845

[39] Su W-C, Cleffi C, Srinivasan S, Bhat A. Telehealth versus face-to-face fine motor and social communication interventions for children with autism spectrum disorder: efficacy, fidelity, acceptability, and feasibility. Am J Occup Ther. 2023;77(6):7706205130. doi: 10.5014/ajot.2023.050282 38048263 PMC10846418

